# Relationship between CYP2C19 Polymorphism and Clopidogrel Resistance in Patients with Coronary Heart Disease and Ischemic Stroke in China

**DOI:** 10.1155/2022/1901256

**Published:** 2022-10-21

**Authors:** Rong Chang, Wenqin Zhou, Yi Ye, Xiaofei Zhang, Yanmin Liu, Jinchun Wu

**Affiliations:** ^1^Department of Cardiology, Shenzhen Longhua District Central Hospital, Shenzhen, China; ^2^The Affiliated Central Hospital of Shenzhen Longhua District, Guangdong Medical University, Shenzhen, Guangdong 518109, China; ^3^Department of Cardiology, Qinghai Provincial People's Hospital, Xining, Qinghai 810007, China

## Abstract

**Objective:**

Clopidogrel is widely used for preventing ischemic complications related to cardiovascular diseases. However, many patients experience clopidogrel resistance (CR). The polymorphisms of CYP2C19 have been implicated in CR, but CYP2C19 polymorphism considerably varies with both ethnic group and geographical location. This study aimed to investigate the association between CYP2C19 polymorphisms and clopidogrel resistance (CR) in patients with coronary heart disease and ischemic stroke among Han and Tibetan populations in Qinghai Province, China.

**Methods:**

From June 2019 to January 2020, patients who were diagnosed with coronary heart disease or cerebral infarction in internal medicine of Qinghai Provincial People's Hospital and had taken dual antiplatelet drugs were included in this study. Blood was collected and routine items were completed. Whole exome sequencing was performed for CYP2C19 genetic polymorphisms of CYP2C19*∗*2 (rs4244285), CYP2C19*∗*3 (rs4986893), and CYP2C19*∗*17 (rs12248560).

**Results:**

A total of 91 patients with coronary heart disease or cerebral infarction (67 Han people (65.99 ± 12.25 years old) and 24 Tibetan (63.6324 Tib years old)) including 52 cases with CR and 39 cases with non-CR were enrolled in this study. For the Han population, the differences in age, glycosylated hemoglobin, activated partial thromboplastin time (APTT), gender, aspirin resistance, and diabetes were significant between the CR and non-CR groups. For the Tibetan population, the two groups showed no significant difference in all indicators. There was no significant difference between CR and non-CR groups for all genotypes (CYP2C19 *∗*2, *∗*3, and *∗*17) in either Han or Tibetan populations. For the Han populations, age, APTT, and aspirin resistance were significantly correlated with CR.

**Conclusion:**

The present study indicated that CYP2C19*∗*2, CYP2C19*∗*3, and CYP2C19*∗*17 alleles were not correlated with CR for both Han and Tibetan populations in Qinghai Province, while age, APTT, and aspirin resistance were independent risk factors of CR in this region.

## 1. Introduction

Cardiac-cerebral disease is common in clinical practice and is the leading cause of long-term disability and death around the world [1]. The global challenges of cardiac-cerebral diseases present an enormous health burden. Coronary heart disease and ischemic stroke are two common cardiac-cerebral diseases, which demonstrates a high-frequency of emergency department visits to manage acute and chronic symptoms [2].

Antiplatelet therapy with aspirin and clopidogrel is frequently used for the secondary prevention of acute coronary syndrome, ischemic stroke, and other related ischemic cardiac-cerebral diseases to reduce recurrent ischemic events [3, 4]. Clopidogrel is an irreversible P2Y12 inhibitor, which is usually used for preventing ischemic complications related to cardiovascular diseases [5]. However, many patients experience relapse or bleeding [6], which is associated with increased late mortality [7, 8]. Clopidogrel resistance (CR), defined as a reduction in platelet aggregation rate by less than 10% from baseline after clopidogrel treatment [9], is considered to be critically associated with recurrent events after anti-platelet therapy.

Studies have reported that the potential mechanisms underlying the insufficient responses to clopidogrel involve several aspects, including epigenetic dysfunction (such as improper DNA methylation), clearance of active metabolites, variable absorption of precursor drugs, potential interactions between drugs, genetic polymorphisms of platelet receptors, adenosine diphosphate (ADP)-mediated variability of P2Y12 platelet receptor, and differences in signal transduction pathway of platelet [10–12]. Additionally, clopidogrel is a prodrug that should be converted into active metabolites by hepatic metabolism through cytochrome (CYP) P450 isoenzymes, including CYP1A2, CYP2C9, CYP2C19, CYP3A, and CYP2B6 [13, 14]. Among these enzymes above, CYP2C19 plays the most important role in clopidogrel transformation [6]. The polymorphisms of CYP2C19 have been demonstrated to be implicated in CR [15, 16]. A recent study demonstrated that the CYP2C19*∗*2 or CYP2C19*∗*3 alleles were significantly related to attenuated platelet response to clopidogrel and increased CR risk among Chinese patients in the Beijing district. Nevertheless, CYP2C19 polymorphism considerably varies with both ethnic group and geographical location [17, 18].

In this study, we intended to investigate the association of CYP2C19 polymorphisms with CR in patients with coronary heart disease and ischemic stroke among Han and Tibetan populations in Qinghai Province, China. The results may help to guide rational clinical drug use and reduce the incidence of cardiovascular adverse events.

## 2. Materials and Methods

### 2.1. Subjects

From June 2019 to January 2020, a total of 91 patients who were diagnosed with coronary heart disease or cerebral infarction in internal medicine of Qinghai Provincial People's Hospital and had taken dual antiplatelet drugs were included in this study. Among them, 67 patients were ethnic Han and 24 were ethnic Tibetan. This study was approved by the Ethics Committee of Qinghai Provincial People's Hospital. All participants had signed the informed consent.

### 2.2. Inclusion and Exclusion Criteria

The inclusion of patients with coronary heart disease was in line with the “coronary heart disease guidelines and expert consensus” in 2019, and patients with stroke or transient ischemic attacks met the “practical diagnosis and expert consensus of ischemic stroke in China” in 2020. The patients were permanent residents or long-time (10 years or more) residents in the Qinghai region. All patients received routine doses of aspirin (100 mg once daily) and clopidogrel (75 mg once daily) for 5–7 days.

The patients with one or more of the following conditions were excluded: allergic or intolerant; contraindications of antiplatelets therapy; rupture and defect of gastrointestinal mucous membrane; inflammation of endocardium or heart valve due to microbial invasion of the body; serious decline in the ability of glomeruli to expel toxins and waste; continuous growth of cancer cells; decreased ejection fraction; combined with pulmonary congestion and inadequate peripheral perfusion with contemplated surgical operation; severe liver disease and/or abnormal coagulation function; and incomplete clinical case data.

### 2.3. Sample Collection, DNA Extraction, and Whole Exome Sequencing

Blood was collected on an empty stomach the next morning after admission for patients who met the inclusion and exclusion criteria, and routine items such as blood routine, biochemistry, thyroid function, and saccharification were completed. Basic clinical data, such as previous history, medication history, personal history, and others, were collected.

A total of 4 ml fasting venous blood was taken and placed in an anticoagulant tube containing ethylenediamine tetraacetic acid (EDTA), and the specimen was stored at −80°C. DNA was extracted using a TianGen DNA extraction kit (TianGen Ltd, Beijing, China). The DNA concentration was determined by fluorescence quantification. Genomic DNA (l *μ*g) was sheared by sonication, and the fragments with an average size of 150–250 bp were selected by magnetic beads. Then, the fragments were ligated to adapters and amplified by pre-PCR. PCR products were then hybridized and washed with Agilent SureSelect or BGI Hybridization and Wash kits (Agilent, CA, USA), followed by PCR amplification. The reaction conductions were 18 cycles of 98°C for 10 s, 65 C for 30 s, and 72°C for 30 s, followed by a final incubation at 72°C for 5 min, and then hold at 4°C. The primers were CYP2C19*∗*2: forward 5′-ATT ACAACCAGAGCTTGGCAT-3′, reverse 5′-GTTGATGTCCATCGATTCTTG-3′; CYP2C19*∗*3: forward 5′-CTGCAATGTGATCTGCTCCAT-3′, reverse 5′-TTCAGGGCTTGGTCAATATAG-3′; and CYP2C19*∗*17: forward 5′-GATGAATGTGGTATATATTCA-3′, reverse 5′-GAGAACAGGACACCTGTTGGT-3′. The library concentration was measured using the Qubit kit (Invitrogen, USA). Sequencing was performed by the combinatorial probe-anchor synthesis method [19]. CYP2C19 genetic polymorphisms of CYP2C19*∗*2 (rs4244285), CYP2C19*∗*3 (rs4986893), and CYP2C19*∗*17 (rs12248560) were recorded.

### 2.4. Statistical Analysis

Statistical analysis was performed using SPSS 22.0 (IBM, Armonk, New York, USA). Quantitative data were assessed for normality by the Shapiro–Wilk test [20]. If the data were normally distributed, they were expressed as the mean ± standard deviation, and the differences were compared by independent sample *t*-test. On the contrary, the data were expressed as median (interquartile range) and the differences were analyzed by Mann–Whitney Test [21]. Qualitative data were represented in the form of N (%) and the difference between groups was analyzed by chi-square test [22]. *P* < 0.05 was considered significant.

## 3. Results

### 3.1. Baseline Information

Finally, 91 patients (67 Han people and 24 Tibetan) meeting the inclusion and exclusion criteria were enrolled in this study. Patient demographics, and clinical and laboratory findings are shown in Table 1. Except for prothrombin time, urea nitrogen, and smoking history (*P* < 0.05), there was no significant difference in the other detection indexes between ethnic Han and Tibetan.

For all patients, according to the definition of CR, there were 52 cases with CR and 39 cases with non-CR. The differences between CR and non-CR groups were significant for age, glycosylated hemoglobin (HBALC), activated partial thromboplastin time (APTT), gender, aspirin resistance, diabetes, and coronary heart disease classification (*P* < 0.05), and the other indicators were not significantly different between the two groups (Table 2).

For the Han population, the differences in age, HBALC, APTT, gender, aspirin resistance, and diabetes were statistically significant between CR and non-CR groups (*P* < 0.05), and the differences in the other indicators were not significant (Table 3). For the Tibetan population, the two groups showed no significant difference in all indicators (Table 4).

### 3.2. Comparative Analysis of CYP2C19 *∗*2 and *∗*3 Loci in Different Ethnic Groups

The genotypes of the CYP2C19*∗*2 locus in all participants included GG (67.0%), GA (26.4%), and AA (6.6%), respectively. The genotypes of the CYP2C19*∗*3 locus were GG (48.4%), GA (26.6%), and AA (23.0%), respectively. The genotypes of the CYP2C19*∗*17 locus were GG and GA, accounting for 65.1% and 34.9%, respectively. For the Han population, the genotypes of the CYP2C19*∗*2 locus were GG (67.2%), GA (23.9%), and AA (9.0%), respectively. The genotypes of CYP2C19*∗*3 locus were GG (50.7%), GA (25.4%), and AA (23.9%), respectively. The genotypes of the CYP2C19*∗*17 locus were GG and GA, accounting for 65.7% and 34.3%, respectively. For the Tibetan population, there were 66.7% GG and 33.3% GA for the genotypes of CYP2C19*∗*2, respectively. The genotypes of the CYP2C19*∗*3 locus were GG (41.7%), GA (37.5%), and AA (20.8%), respectively. For the genotypes of the CYP2C19*∗*17 locus, GG accounted for 54.2 and GA accounted for 45.8% (Table 1). There was no significant difference between ethnic Han and Tibetan.

### 3.3. Comparative Analysis of Different Locus between CR and Non-CR Groups

For all patients, the genotypes of CYP2C19*∗*2, CYP2C19*∗*3, and CYP2C19*∗*17 for CR and non-CR groups are shown in Table 2. The genotypes of CYP2C19*∗*2, CYP2C19*∗*3, and CYP2C19*∗*17 for the Han population in two groups are shown in Table 3 and for the Tibetan population in two groups are shown in Table 4. There was no significant difference between CR and non-CR groups for all genotypes in either Han or Tibetan populations.

### 3.4. Logistic Analysis of Risk Factors for CR

Variables with significant differences in baseline information were included for univariate and multivariate logistic regression analyses to explore the significant related factors of CR. As shown in Table 5, for all patients, age was a significant risk factor for CR, with an odds ratio (OR) (95% confidence interval (CI)) = 1.08 (1.02, 1.13), *P*=0.005. The older the patients, the higher the risk of CR. APTT was a significant negative correlation factor for CR (OR (95% CI) = 0.81 (0.69, 0.95), *P*=0.011), and the risk of CR decreased with the increase of APTT. Aspirin resistance was a significant positive correlation factor for CR (OR (95% CI) = 6.47 (2.02, 20.67), *P*=0.002). Patients with aspirin resistance were at a significantly increased risk of developing CR. There was no significant association between coronary heart disease type and CR. For the Han populations, age, APTT, and aspirin resistance were significantly correlated with CR (*P* < 0.05) (Table 6).

## 4. Discussion

Clopidogrel combined with aspirin is usually recommended for preventing ischemic events in patients with cardiovascular [23]. Despite the standard treatment, there are still a lot of adverse cardiovascular events, and CR is considered to be the main reason [24]. In this study, we investigated the association between *∗*2, *∗*3, and *∗*17 allelic variants of the CYP2C19 gene and CR in patients with coronary heart disease and ischemic stroke among Han and Tibetan populations. The results showed that three alleles were not statistically correlated with CR, while age, APTT, and aspirin resistance were significantly correlated with CR.

Presently, the mechanisms underlying CR have not been fully elucidated. The CYP2C19 genotype is the most important determinant of the pharmacodynamic and pharmacokinetic responses to clopidogrel [25]. It has been reported that CYP2C19*∗*2 and CYP2C19*∗*3, the main mutant alleles, are the most common genotypes in Asian populations [26]. CYP2C19*∗*2 or CYP2C19*∗*3 allelic variants increase the risk of CR [27]. CYP2C19*∗*17 allele is correlated with an increased risk of bleeding [28]. However, the present study showed that CYP2C19*∗*2, CYP2C19*∗*3, and CYP2C19*∗*17 alleles were not significantly different between Han or Tibetan populations as well as between CR and non-CR groups, which suggested that the three alleles were not statistically correlated with CR in this study.

Our result was in accordance with a recent study that investigated the association between CYP2C19*∗*2, CYP2C19*∗*3, and CYP2C19*∗*17 variants of the CYP2C19 gene and CR in patients with acute coronary syndromes in Morocco, and demonstrated that none of the three alleles showed a statistical correlation with CR. Different from the results of our study, that study identified a synergic effect among the three alleles on CR [29]. In fact, the correlation between polymorphisms of CYP2C19 and platelet responsiveness to clopidogrel has been widely recognized among patients with acute coronary syndrome and percutaneous coronary intervention, but the association with other indications, such as arterial fibrillation and stable angina, is negative [30, 31]. The inconsistent results may be due to the magnitude of the influence of CYP2C19 on the effectiveness of clopidogrel and may be consistent with the influence of this molecule on specific clinical indications [32, 33].

In this study, APTT, age, and aspirin resistance were significantly correlated with CR. The APTT is a widely available test used to screen for hypercoagulable states in bleeding disorders [34]. Shortened APTT is an independent risk factor for ischemic stroke [35], but its role in CR has not been reported to our knowledge. Age was a positively correlated factor of CR, which was inconsistent with previous studies. Prabhakaran et al. [36] have reported that being older than 55 years contributed to a low response to clopidogrel loading. It has been reported that patients with aspirin resistance have increased platelet reactivity [37]. High on-treatment platelet reactivity has become the most important factor inhibiting the antiplatelet effect of clopidogrel, resulting in the ineffectiveness of this agent [38]. Clopidogrel's high on-treatment platelet reactivity could negatively influence the clinical course of a stroke and increase the risk of recurrent vascular events [39]. Therefore, platelet function testing is necessary for stroke individuals, especially those predisposed to CR.

There were several limitations in the present study. First, there was a lack of sequence analysis that could provide more robust information on the investigated CYP2C19 polymorphisms. Second, the study only comprised Chinese patients, while multicentric investigation might have been more informative in terms of data robustness. Third, there was a lack of functional correlation between examined gene polymorphisms and enzyme activity in patients. At last, no control group represented by healthy individuals was included in the analysis. Furthermore, studies including larger sample sizes and control groups may help to better understand the phenomenon of heterogeneity in clopidogrel response.

## 5. Conclusion

In conclusion, the present study indicated that CYP2C19*∗*2, CYP2C19*∗*3, and CYP2C19*∗*17 alleles were not correlated with CR for both Han and Tibetan populations in Qinghai Province, while age, APTT, and aspirin resistance were independent risk factors of CR in this region. Our results may provide useful data for precision medicine based on individual gene sequencing results.

## Figures and Tables

**Table 1:** Baseline information for participants.

Characteristics	Total (*N* = 91)	Han population (*N* = 67)	Tibetan population (*N* = 24)	*P* value
Age, years^#^	65.36 ± 12.44	65.99 ± 12.25	63.63 ± 13.06	0.428
Height (m)^#^	1.68 ± 0.08	1.68 ± 0.09	1.66 ± 0.08	0.251
Weight (Kg)^#^	68.12 ± 10.75	68.88 ± 11.05	66.00 ± 9.80	0.262
BMI (Kg/m^2^)^#^	24.11 ± 2.71	24.19 ± 2.74	23.91 ± 2.68	0.663
Oxygen saturation (%)	90.0 (89.0, 93.0)	91.0 (89.0, 93.0)	90.0 (90.0, 92.0)	0.816
SBP (mmHg)^#^	126.9 ± 24.4	128.7 ± 22.3	122.1 ± 29.4	0.328
DBP (mmHg)^#^	74.0 ± 13.5	73.9 ± 12.3	74.1 ± 16.8	0.960
Heart rate^#^	76.8 ± 15.5	78.5 ± 15.0	72.1 ± 16.2	0.080
White blood cell	7.20 (5.70, 11.09)	7.05 (5.55, 10.96)	7.88 (6.64, 11.33)	0.471
Red blood cell	4.77 (4.26, 5.31)	4.75 (4.40, 5.39)	4.78 (4.03, 5.22)	0.365
Hemoglobin^#^	151.1 ± 24.2	153.2 ± 21.5	145.0 ± 30.2	0.156
Glycosylated hemoglobin	5.81 (5.47, 6.70)	5.78 (5.46, 6.70)	5.95 (5.59, 6.95)	0.418
PT	12.0 (11.5, 12.6)	11.9 (11.4, 12.5)	12.2 (11.9, 13.4)	0.010
APTT	27.6 (25.4, 31.2)	27.5 (25.4, 30.8)	30.7 (25.9, 32.8)	0.072
ALT	30.0 (19.0, 54.0)	29.0 (19.0, 54.0)	38.0 (17.0, 66.8)	0.496
AST	38.0 (22.0, 159.0)	34.0 (21.0, 159.0)	44.5 (22.3, 258.5)	0.438
AST/ALT	1.2 (0.8, 2.4)	1.3 (0.9, 2.8)	1.1 (0.7, 2.1)	0.355
Urea nitrogen	6.07 (4.98, 8.10)	5.87 (4.97, 7.58)	7.16 (5.99, 10.68)	0.014
Creatinine	84.0 (67.0, 94.0)	82.0 (67.0, 93.0)	84.5 (75.3, 114.5)	0.242
Glomerular filtration rate (mL/min × 1.73 m^2^)#	78.2 ± 25.2	80.6 ± 24.9	70.9 ± 25.1	0.109
Albumin	37.8 (34.8, 40.6)	38.0 (35.2, 40.7)	36.6 (32.7, 38.3)	0.085
Total cholesterol (mmol/L)^#^	4.13 ± 1.12	4.13 ± 1.03	4.02 ± 1.37	0.699
Triglyceride	1.42 (0.90, 2.14)	1.59 (0.90, 2.18)	1.32 (0.88, 1.87)	0.418
TSH	1.60 (0.86, 2.82)	1.62 (0.85, 2.70)	1.30 (0.88, 2.91)	0.571

Sex, *n* (%)				0.475
Male	63 (69.2)	45 (67.2)	18 (75.0)	
Female	28 (30.8)	22 (32.8)	6 (25.0)	

Aspirin resistance, *n* (%)				0.660
No	42 (46.2)	30 (44.8)	12 (50.0)	
Yes	49 (53.8)	37 (55.2)	12 (50.0)	

Smoking history, *n* (%)				0.039
No	52 (57.1)	34 (50.7)	18 (75.0)	
Yes	39 (42.9)	33 (49.3)	6 (25.0)	

Coronary heart disease type, *n* (%)				0.932
STEMI	42 (46.2)	31 (46.3)	11 (45.8)	
NSTEMI	12 (13.2)	8 (11.9)	4 (16.7)	
SAP	15 (16.5)	11 (16.4)	4 (16.7)	
UA	22 (24.2)	17 (25.4)	5 (20.8)	

Hypertension, *n* (%)				0.291
No	30 (33.0)	20 (29.9)	10 (41.7)	
Yes	61 (66.7)	47 (70.1)	14 (58.3)	

Diabetes, *n* (%)				0.374
No	45 (49.5)	35 (52.2)	10 (41.7)	
Yes	46 (50.5)	32 (47.8)	14 (58.3)	

Stroke, *n* (%)				0.473
No	59 (64.8)	42 (62.7)	17 (70.8)	
Yes	32 (35.2)	25 (37.3)	7 (29.2)	

Hyperlipidaemia, *n* (%)				0.907
No	54 (59.3)	40 (59.7)	14 (58.3)	
Yes	37 (40.7)	27 (40.3)	10 (41.7)	

CYP2C19*∗*2, *n* (%)				0.120
GG	61 (67.0)	45 (67.2)	16 (66.7)	
GA	24 (26.4)	16 (23.9)	8 (33.3)	
AA	6 (6.6)	6 (9.0)	0 (0.0)	

CYP2C19*∗*3, *n* (%)				0.527
GG	44 (48.4)	34 (50.7)	10 (41.7)	
GA	26 (28.6)	17 (25.4)	9 (37.5)	
AA	21 (23.0)	16 (23.9)	5 (20.8)	

CYP2C19*∗*17, *n* (%)				0.317
GG	57 (62.6)	44 (65.7)	13 (54.2)	
GA	34 (37.4)	23 (34.3)	11 (45.8)	

^#^mean ± sd, *P* value, Han population vs. Tibetan population.

**Table 2:** Comparison of differences in baseline information between CR and non-CR for all participants.

Characteristics	Non-CR (*N* = 39)	CR (*N* = 52)	*P* value
Age, years^#^	59.46 ± 11.01	69.79 ± 11.67	<0.001
Height (m)^#^	1.70 ± 0.08	1.66 ± 0.09	0.062
Weight (Kg)^#^	68.56 ± 9.64	67.79 ± 11.60	0.736
BMI (Kg/m^2^)^#^	23.71 ± 2.43	24.41 ± 2.89	0.225
Oxygen saturation (%)	91.0 (89.0, 93.0)	90.0 (90.0, 92.8)	0.827
SBP (mmHg)^#^	126.3 ± 27.2	127.4 ± 22.3	0.837
DBP (mmHg)^#^	74.9 ± 14.9	73.2 ± 12.5	0.553
Heart rate^#^	76.5 ± 14.8	77.1 ± 16.2	0.854
White blood cell	7.32 (5.55, 10.06)	7.17 (5.71, 11.33)	0.721
Red blood cell	4.89 (4.40, 5.54)	4.70 (4.25, 5.22)	0.342
Hemoglobin^#^	153.2 ± 28.7	149.5 ± 20.3	0.500
Glycosylated hemoglobin	5.63 (5.38, 6.19)	6.06 (5.52, 7.17)	0.005
PT	12.0 (11.7, 12.7)	11.9 (11.4, 12.5)	0.271
APTT	28.7 (26.7, 32.3)	26.9 (24.3, 31.0)	0.008
ALT	37.0 (19.0, 82.0)	28.5 (19.3, 42.0)	0.183
AST	48.0 (22.0, 221.0)	33.5 (21.3, 109.5)	0.195
AST/ALT	1.3 (0.9, 3.4)	1.2 (0.8, 2.3)	0.431
Urea nitrogen	5.91 (4.97, 10.04)	6.28 (5.25, 7.99)	0.745
Creatinine	83.0 (67.0, 96.0)	84.0 (67.3, 92.8)	0.591
Glomerular filtration rate (mL/min × 1.73 m^2^)^#^	80.7 ± 28.3	76.0 ± 22.7	0.389
Albumin	38.2 (34.7, 41.1)	37.8 (34.9, 39.7)	0.510
Total cholesterol (mmol/L)^#^	4.00 ± 1.22	4.17 ± 1.05	0.478
Triglyceride	1.32 (0.90, 1.97)	1.59 (0.90, 2.38)	0.432
TSH	1.19 (0.54, 2.68)	1.68 (1.20, 2.89)	0.156

Sex, *n* (%)			0.022
Male	32 (82.1)	31 (59.6)	
Female	7 (17.9)	21 (40.4)	

Aspirin resistance, *n* (%)			<0.001
No	28 (71.8)	14 (26.9)	
Yes	11 (28.2)	38 (73.1)	

Smoking history, *n* (%)			0.328
No	20 (51.3)	32 (61.5)	
Yes	19 (48.7)	20 (38.5)	

Coronary heart disease type, *n* (%)			0.041
STEMI	19 (48.7)	23 (44.2)	
NSTEMI	8 (20.5)	4 (7.7)	
SAP	2 (5.1)	13 (25.0)	
UA	10 (25.6)	12 (23.1)	

Hypertension, *n* (%)			0.157
No	16 (41.0)	14 (26.9)	
Yes	23 (59.0)	38 (73.1)	

Diabetes, *n* (%)			0.004
No	26 (66.7)	19 (36.5)	
Yes	13 (33.3)	33 (63.5)	

Stroke, *n* (%)			0.899
No	25 (64.1)	34 (65.4)	
Yes	14 (35.9)	18 (34.6)	

Hyperlipidaemia, *n* (%)			0.355
No	21 (53.8)	33 (63.5)	
Yes	18 (46.2)	19 (36.5)	

CYP2C19*∗*2, *n* (%)			0.412
GG	24 (61.5)	37 (71.2)	
GA	11 (28.2)	13 (25.0)	
AA	4 (10.3)	2 (3.8)	

CYP2C19*∗*2, *n* (%)			0.334
GG	24 (61.5)	37 (71.2)	
GA + AA	15 (38.5)	15 (28.8)	

CYP2C19*∗*3, *n* (%)			0.387
GG	22 (56.4)	22 (42.3)	
GA	10 (25.6)	16 (30.8)	
AA	7 (17.9)	14 (26.9)	

CYP2C19*∗*3, *n* (%)			0.183
GG	22 (56.4)	22 (42.3)	
GA + AA	17 (43.6)	30 (57.7)	

CYP2C19*∗*17, *n* (%)			0.532
GG	23 (59.0)	34 (65.4)	
GA	16 (41.0)	18 (34.6)	

**Table 3:** Comparison of differences in baseline information between CR and non-CR for Han populations.

Characteristics	Non-CR (*N* = 27)	CR (*N* = 40)	*P* value
Age, years^#^	59.33 ± 10.48	70.48 ± 11.38	<0.001
Height (m)^#^	1.70 ± 0.08	1.67 ± 0.09	0.115
Weight (Kg)^#^	69.04 ± 9.93	68.78 ± 11.86	0.925
BMI (Kg/m^2^)^#^	23.60 ± 2.13	24.59 ± 3.04	0.151
Oxygen saturation (%)	91.0 (89.0, 93.0)	90.5 (89.3, 93.0)	0.892
SBP (mmHg)^#^	129.6 ± 28.1	128.1 ± 17.7	0.809
DBP (mmHg)^#^	75.0 ± 14.1	73.2 ± 11.0	0.549
Heart rate^#^	77.7 ± 14.2	79.1 ± 15.7	0.699
White blood cell	7.17 (5.55, 10.53)	6.93 (5.48, 11.06)	0.898
Red blood cell	5.10 (4.55, 5.55)	4.60 (4.25, 5.26)	0.120
Hemoglobin^#^	158.5 ± 22.1	149.7 ± 20.6	0.098
Glycosylated hemoglobin	5.57 (5.38, 5.97)	6.09 (5.49, 7.24)	0.009
PT	11.9 (11.6, 12.7)	11.7 (11.2, 12.5)	0.169
APTT	27.9 (26.7, 30.9)	26.5 (24.5, 30.0)	0.031
ALT	33.0 (20.0, 70.0)	27.0 (18.3, 39.5)	0.125
AST	60.0 (22.0, 209.0)	31.0 (20.0, 121.5)	0.179
AST/ALT	1.4 (1.0, 3.4)	1.2 (0.8, 2.4)	0.315
Urea nitrogen	5.41 (4.86, 7.54)	6.11 (5.25, 7.63)	0.201
Creatinine	78.0 (67.0, 90.0)	84.5 (67.0, 93.0)	0.908
Glomerular filtration rate (mL/min × 1.73 m^2^)^#^	87.4 ± 28.3	75.9 ± 21.5	0.064
Albumin	38.6 (36.1, 41.4)	37.8 (35.2, 40.0)	0.457
Total cholesterol (mmol/L)^#^	4.07 ± 1.02	4.16 ± 1.04	0.731
Triglyceride	1.29 (0.90, 1.97)	1.77 (0.90, 2.51)	0.315
TSH	1.79 (0.66, 2.65)	1.62 (1.15, 2.81)	0.498

Sex, *n* (%)			0.010
Male	23 (85.2)	22 (55.0)	
Female	4 (14.8)	18 (45.0)	

Aspirin resistance, *n* (%)			<0.001
No	20 (74.1)	10 (25.0)	
Yes	7 (25.9)	30 (75.0)	

Smoking history, *n* (%)			0.065
No	10 (37.0)	24 (60.0)	
Yes	17 (63.0)	16 (40.0)	

Coronary heart disease type, *n* (%)			0.206
STEMI	14 (51.9)	17 (42.5)	
NSTEMI	5 (18.5)	3 (7.5)	
SAP	2 (7.4)	9 (22.5)	
UA	6 (22.2)	11 (27.5)	

Hypertension, *n* (%)			0.110
No	11 (40.7)	9 (22.5)	
Yes	16 (59.3)	31 (77.5)	

Diabetes, *n* (%)			0.003
No	20 (74.1)	15 (37.5)	
Yes	7 (25.9)	25 (62.5)	

Stroke, *n* (%)			0.969
No	17 (63.0)	25 (62.5)	
Yes	10 (37.0)	15 (37.5)	

Hyperlipidaemia, *n* (%)			0.570
No	15 (55.6)	25 (62.5)	
Yes	12 (44.4)	15 (37.5)	

CYP2C19*∗*2, *n* (%)			0.333
GG	16 (59.3)	29 (72.5)	
GA	7 (25.9)	9 (22.5)	
AA	4 (14.8)	2 (5.0)	

CYP2C19*∗*2, *n* (%)			0.258
GG	16 (59.3)	29 (72.5)	
GA + AA	11 (40.7)	11 (27.5)	

CYP2C19*∗*3, *n* (%)			0.216
GG	17 (63.0)	17 (42.5)	
GA	6 (22.2)	11 (27.5)	
AA	4 (14.8)	12 (30.0)	

CYP2C19*∗*3, *n* (%)			0.100
GG	17 (63.0)	17 (42.5)	
GA + AA	10 (37.0)	23 (57.5)	

CYP2C19*∗*17, *n* (%)			0.888
GG	18 (66.7)	26 (65.0)	
GA	9 (33.3)	14 (35.0)	

**Table 4:** Comparison of differences in baseline information between CR and non-CR for Tibetan populations.

Characteristics	Non-CR (*N* = 12)	CR (*N* = 12)	*P* value
Age, years^#^	59.75 ± 12.63	67.50 ± 12.82	0.150
Height (m)^#^	1.68 ± 0.08	1.64 ± 0.08	0.227
Weight (Kg)^#^	67.50 ± 9.26	64.50 ± 10.49	0.466
BMI (Kg/m^2^)^#^	23.97 ± 3.11	23.84 ± 2.31	0.911
Oxygen saturation (%)	90.5 (89.0, 92.8)	90.0 (90.0, 92.0)	0.630
SBP (mmHg)^#^	119.1 ± 24.6	125.2 ± 34.4	0.623
DBP (mmHg)^#^	74.8 ± 17.3	73.4 ± 17.1	0.851
Heart rate^#^	73.8 ± 16.4	70.3 ± 16.5	0.607
White blood cell	7.35 (5.36, 9.55)	9.08 (6.86, 15.20)	0.266
Red blood cell	4.55 (3.78, 5.49)	4.79 (4.19, 5.19)	0.731
Hemoglobin^#^	141.1 ± 38.3	149.0 ± 20.1	0.533
Glycosylated hemoglobin	5.69 (5.26, 7.04)	6.01 (5.81, 6.91)	0.291
PT	12.1 (11.9, 13.3)	12.3 (11.9, 13.5)	0.799
APTT	31.8 (27.2, 35.0)	29.4 (23.6, 32.0)	0.114
ALT	45.0 (12.0, 134.8)	35.5 (20.3, 51.8)	0.887
AST	43.0 (20.0, 771.8)	44.5 (23.3, 106.0)	0.843
AST/ALT	1.0 (0.7, 4.7)	1.1 (0.6, 2.1)	0.887
Urea nitrogen	8.56 (5.99, 13.66)	7.16 (5.13, 8.80)	0.443
Creatinine	93.5 (76.3, 127.5)	82.5 (72.8, 88.5)	0.319
Glomerular filtration rate (mL/min × 1.73 m^2^)^#^	65.5 ± 22.6	76.4 ± 27.3	0.297
Albumin	37.7 (32.6, 41.0)	36.2 (33.2, 38.3)	0.833
Total cholesterol (mmol/L)^#^	3.84 ± 1.62	4.20 ± 1.10	0.528
Triglyceride	1.38 (0.92, 1.95)	1.16 (0.89, 2.09)	0.799
TSH	1.12 (0.36, 2.94)	2.04 (1.26, 2.90)	0.211

Sex, *n* (%)			1.000
Male	9 (75.0)	9 (75.0)	
Female	3 (25.0)	3 (25.0)	

Aspirin resistance, *n* (%)			0.102
No	8 (66.7)	4 (33.3)	
Yes	4 (33.3)	8 (66.7)	

Smoking history, *n* (%)			0.342
No	10 (83.3)	8 (66.7)	
Yes	2 (16.7)	4 (33.3)	

Coronary heart disease type, *n* (%)			0.618
STEMI	5 (41.7)	6 (50.0)	
NSTEMI	3 (25.0)	1 (8.3)	
SAP	0 (0.0)	4 (33.3)	
UA	4 (33.3)	1 (8.3)	

Hypertension, *n* (%)			1.000
No	5 (41.7)	5 (41.7)	
Yes	7 (58.3)	7 (58.3)	

Diabetes, *n* (%)			0.408
No	6 (50.0)	4 (33.3)	
Yes	6 (50.0)	8 (66.7)	

Stroke, *n* (%)			0.653
No	8 (66.7)	9 (75.0)	
Yes	4 (33.3)	3 (25.0)	

Hyperlipidaemia, *n* (%)			0.408
No	6 (50.0)	8 (66.7)	
Yes	6 (50.0)	4 (33.3)	

CYP2C19*∗*2, *n* (%)			1.000
GG	8 (66.7)	8 (66.7)	
GA	4 (33.3)	4 (33.3)	

CYP2C19*∗*3, *n* (%)			0.855
GG	5 (41.7)	5 (41.7)	
GA	4 (33.3)	5 (41.7)	
AA	3 (25.0)	2 (16.7)	

CYP2C19*∗*3, *n* (%)			1.000
GG	5 (41.7)	5 (41.7)	
GA + AA	7 (58.3)	7 (58.3)	

CYP2C19*∗*17, *n* (%)			0.219
GG	5 (41.7)	8 (66.7)	
GA	7 (58.3)	4 (33.3)	

**Table 5:** Univariate and multivariate logistics regression analyses for CR in all participants.

Variables	Univariate analysis		Multivariate analysis	
	OR (95% CI)	*P* value	OR (95% CI)	*P* value
Age	1.08 (1.04, 1.12)	<0.001	1.08 (1.02, 1.13)	0.005
Glycosylated hemoglobin	2.00 (1.19, 3.39)	0.010	Excluded	
APTT	0.86 (0.78, 0.96)	0.006	0.81 (0.69, 0.95)	0.011
Sex			Excluded	
Male	Reference			
Female	3.10 (1.15, 8.32)	0.025		
Aspirin resistance				
No	Reference		Reference	
Yes	6.91 (2.73, 17.48)	<0.001	6.47 (2.02, 20.67)	0.002
Diabetes			Excluded	
No	Reference			
Yes	3.47 (1.45, 8.32)	0.005		
Coronary heart disease type, *n* (%)		0.073		0.053
STEMI	Reference		Reference	
NSTEMI	0.41 (0.11, 1.59)	0.198	0.19 (0.03, 1.12)	0.066
SAP	5.37 (1.08, 26.81)	0.040	4.43 (0.57, 34.57)	0.156
UA	0.99 (0.35, 2.79)	0.987	0.39 (0.10, 1.55)	0.181

**Table 6:** Univariate and multivariate logistics regression analyses for CR in Han populations.

Variables	Univariate analysis		Multivariate analysis	
	OR (95% CI)	*P* value	OR (95% CI)	*P* value
Age	1.09 (1.04, 1.15)	0.001	1.09 (1.03, 1.15)	0.003
Glycosylated hemoglobin	2.37 (1.16, 4.87)	0.019	Excluded	
APTT	0.86 (0.76, 0.98)	0.027	0.82 (0.67, 1.00)	0.048

Sex			Excluded	
Male	Reference			
Female	4.71 (1.37, 16.11)	0.014		

Aspirin resistance				
No	Reference		Reference	
Yes	8.57 (2.80, 26.25)	<0.001	7.92 (2.14, 29.36)	0.002

Diabetes			Excluded	
No	Reference			
Yes	4.76 (1.63, 13.92)	0.004		

## Data Availability

The data used to support the findings of this study are available from the corresponding author upon request.
